# Open surgical repair of traumatic abdominal aortic dissection with superior mesenteric arterial occlusion

**DOI:** 10.1016/j.jvscit.2025.102000

**Published:** 2025-11-04

**Authors:** Hung Quoc Doan, Son Duy Hong Phung, Hiep Xuan Vu, Hung Duc Duong, Hien Thi Thu Do, Thang Ngoc Duong

**Affiliations:** a Cardiovascular Center, Vinmec International Hospital, Hanoi, Viet Nam; b Cardiovascular and Thoracic Center, Viet Duc University Hospital, Hanoi, Viet Nam; c Surgery Department, Hanoi Medical University, Hanoi, Viet Nam; d Department of Urology, Hanoi Medical University Hospital, Hanoi, Viet Nam

**Keywords:** Abdominal aortic trauma, Ischemia mesenteric post-trauma, Superior mesenteric arterial trauma

## Abstract

Traumatic abdominal aortic injury with visceral arterial involvement is rare and associated with high mortality, even with prompt intervention. We present a case of mesenteric ischemia caused by traumatic dissection of the infrarenal abdominal aorta and occlusion of the superior mesenteric artery. The patient underwent open surgical repair with infrarenal aortic replacement and an iliac–mesenteric bypass to restore visceral perfusion. Despite established organ ischemia, classical surgical techniques proved effective. This case highlights the importance of timely diagnosis and aggressive vascular reconstruction in salvaging patients with complex aortic trauma and visceral hypoperfusion. Successful outcomes remain achievable with appropriate surgical intervention.

Blunt traumatic injury to the abdominal aorta is rare, occurring in approximately 5% of patients with aortic injury.^1^ This exceptionally low incidence is attributed both to the protected anatomic position of the abdominal aorta and the high pre-hospital and in-hospital mortality associated with such lesions.^2^^,^^3^ Although uncommon, abdominal aortic injury can be complicated by acute visceral ischemia when the superior mesenteric artery (SMA) is involved.^4^ Disruption of flow may occur secondary to intimal injury, dissection, or thrombotic occlusion, and is associated with rapid progression to bowel infarction if not promptly addressed.^5^ The coexistence of SMA occlusion with traumatic aortic injury significantly worsens prognosis due to the combined hemodynamic insult and the narrow therapeutic window for visceral salvage. This report presents a case of acute intestinal ischemia following blunt trauma to the abdominal aorta.

## Case -reports

A 61-year-old previously healthy male sustained a workplace injury after being pulled into a conveyor belt system. He received initial resuscitation at a provincial hospital and was transferred to our facility 12 hours post-trauma. At the time of admission, the patient was alert and hemodynamically stable. Physical examination revealed chest tenderness with mildly decreased bilateral breath sounds, mild abdominal distension with right upper quadrant tenderness, no peritoneal signs, and lumbar spine tenderness without neurological deficits. The left femoral pulse was absent, and the left lower limb was cold compared with the right, although painless. The only abnormal laboratory finding was an elevated serum lactate level of 8.2 mmol/L.

**Fig 1:**
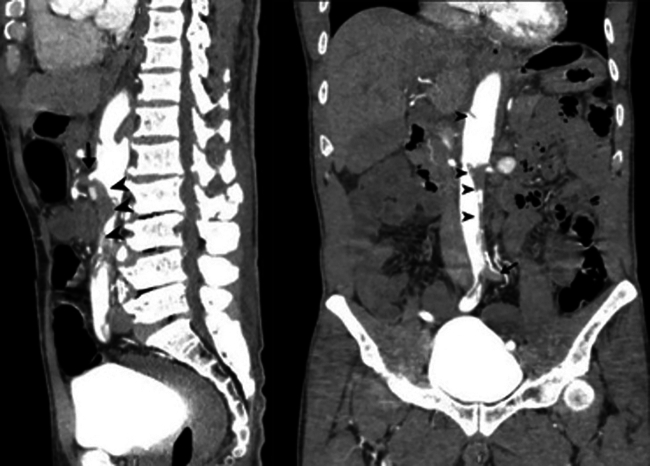
Abdominal aortic injury with superior mesenteric artery (SMA) occlusion in multi-detector computed tomography (*arrowhead*, aortic dissection with multiple entry; *arrow*, SMA total occlusion).

Contrast-enhanced scanner of the chest and abdomen revealed bilateral fractures of the fifth and sixth ribs, a minimally displaced sternal body fracture, bilateral hemothorax, and transverse process fractures of the L1 to L3 vertebrae. Hepatic injury was classified as grade II. A dissection of the abdominal aorta was identified, extending from just below the diaphragm to the aortic bifurcation. The intimal tear was located proximally to the origin of the SMA. The true lumen was larger than the false lumen, which was completely thrombosed at the origin of the SMA and the left common iliac artery, resulting in complete occlusion of both vessels (Fig 1).

**Fig 2:**
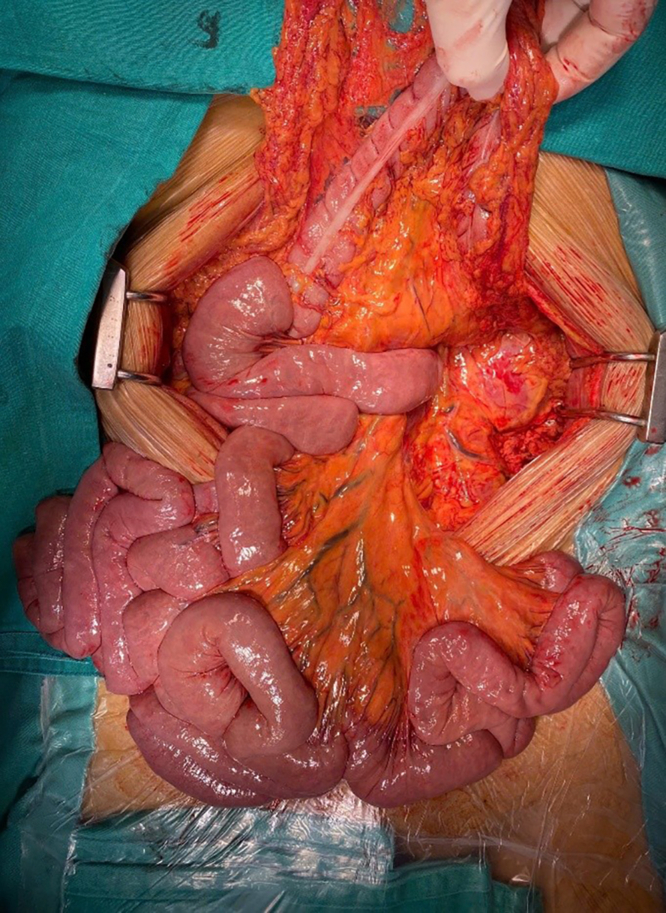
Jejunal, illeal, and colonic acute ischemia.

**Fig 3:**
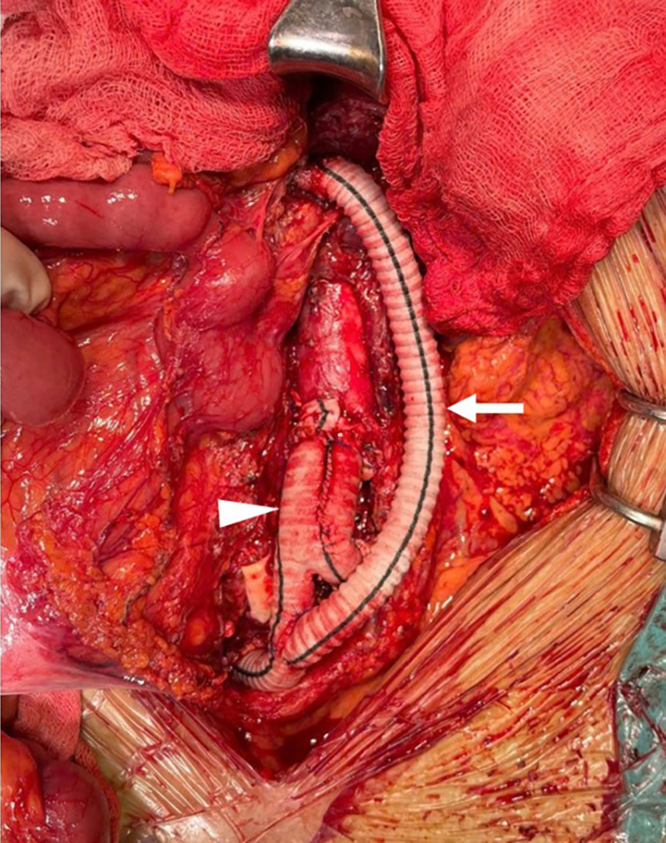
Abdominal aortic replacement (*arrowhead*, aortic prothesis; *arrow*, iliac-superior mesenteric artery [SMA] bypass).

The procedure was performed with collaboration between vascular and digestive surgical teams. Upon exploratory laparotomy, the liver, stomach, small bowel, and colon appeared dusky, with absent peristalsis and reduced tone, consistent with visceral hypoperfusion (Fig 2). Exposure of the infrarenal abdominal aorta revealed a diffusely discolored vessel wall extending to the bifurcation of the right internal-external iliac arteries. The left common iliac artery was completely thrombosed due to compression of the true lumen by the false lumen. The SMA was pulseless, with complete thrombosis extending approximately 2 cm from its origin. A Y-shaped Dacron graft (16 mm × 8 mm) was used to replace the abdominal aorta below the inferior mesenteric artery and bilateral iliac arteries. An intimal fenestration was created to establish flow between the true and false lumens. A bypass from the SMA to the right limb of the graft was constructed using an 8-mm Dacron conduit (Fig 3). After closure of the posterior parietal peritoneum, an omental flap was mobilized to cover the entire vascular graft, serving as a protective barrier to reduce the risk of postoperative infection. A segment of ileum approximately 30 cm proximal to the ileocecal junction and the left colon, which appeared necrotic and dusky due to mesenteric contusion, was resected and followed by exteriorization of both ends.

**Fig 4:**
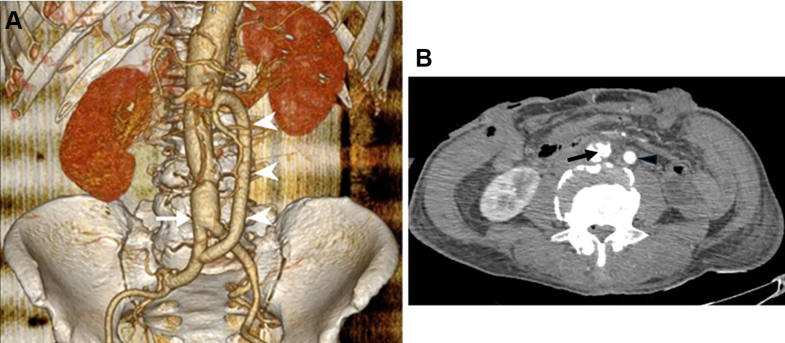
Angiography postoperation. **(A)** Three-dimensional aortic reconstruction; *white arrow* demonstrates the aortic prothesis; *white arrowheads* reveal the iliac-superior mesenteric artery (SMA) bypass. **(B)***Black arrow* demonstrates the intimal fenestration created above the aortic anastomosis; *black arrowhead* reveals the iliac-SMA bypass.

The patient was discharged after 27 days in stable condition. He tolerated oral intake well, with good function of the exteriorized stomas. Bilateral lower limb pulses were palpable. Pre-discharge scan confirmed patency of all vascular grafts and demonstrated adequate contrast enhancement of the abdominal viscera, indicating restored perfusion (Fig 4). The patient provided written informed consent for publication.

## Discussion

Acute visceral ischemia due to SMA injury following trauma is exceedingly rare, accounting for approximately 0.09% of trauma cases, yet poses a significant threat to patient survival. Even with aggressive resuscitation and emergency surgical intervention, postoperative mortality rates exceed 50%.^6^ Previous reports have primarily described direct trauma to the SMA; however, in our case, occlusion resulted from a static mechanism resembling pathological aortic dissection. Clinically, the patient exhibited signs of mucosal bowel necrosis, including hematochezia, elevated serum lactate, and dusky, nonperistaltic bowel loops on exploration. Without timely treatment, the likelihood of mortality is nearly absolute.^7^

In 1972, Fullen et al proposed a classification system for SMA injuries based on anatomical location.^8^ Injuries at the SMA origin were designated as Grade I—the most severe—due to their potential to compromise perfusion to the jejunum, ileum, and right colon. Safaya et al^9^ reported cases of antegrade stent placement to revascularize a traumatically injured superior mesenteric artery. Endovascular techniques offer the advantage of being minimally invasive; however, in our experience, a key limitation is the inability to adequately assess intestinal ischemia both before and after revascularization. Therefore, close postoperative monitoring is essential to avoid delayed bowel resection in cases where necrosis has already occurred. Given the presence of abdominal aortic dissection in our patient, we selected a midline transperitoneal approach. The SMA was exposed approximately 1 cm distal to its origin using anterior transperitoneal exposure, ligament of Treitz was incised to mobilize the fourth portion of duodenum, and the SMA was exposed by incising the peritoneum directly above it at the root of the mesentery. The anastomosis was performed proximally to accommodate the size of the prosthetic graft (8 mm) and to ensure adequate hemodynamic perfusion to the first major branches.

We replaced the segment of the abdominal aorta distal to the origin of the inferior mesenteric artery to preserve collateral mesenteric circulation and minimize disruption of native visceral perfusion pathways. The goals of aortic replacement were two-fold: to restore bilateral lower limb perfusion and to establish a healthy landing zone for the SMA bypass. A major technical challenge was the friability of the dissected aortic wall, which posed a risk of anastomotic tearing and hemorrhage. To reinforce the aortic anastomosis, a felt strip was applied.

Following completion of the aortic-to-graft anastomosis, we observed absent flow from the true lumen into the graft. To address this, an intimal fenestration was created just above the anastomosis to ensure communication between the true and false lumens. This technique, previously described in the management of visceral and limb ischemia due to chronic aortic dissection,^10^ not only restored antegrade flow into the graft but also reduced pressure within the false lumen, potentially lowering the risk of future postdissection aneurysmal degeneration.

The digestive surgical team was routinely involved to assess bowel viability following revascularization and to resect segments exhibiting irreversible ischemia. Postoperative intensive care played a critical role due to the risk of systemic complications, particularly reperfusion injury to the bowel, which can precipitate multiorgan failure and even death.^11^

## Conclusion

Our report highlights the critical importance of early recognition of visceral ischemia due to traumatic occlusion of the SMA, guided by clinical examination and suggestive laboratory findings. Multi-detector computed tomography remains the gold standard for definitive diagnosis and for distinguishing the underlying mechanism of injury, thereby informing appropriate surgical strategies. Even in delayed presentations, thorough assessment of visceral perfusion, systemic status, and individualized operative planning can offer a viable path to survival in patients with complex aorto-mesenteric trauma.

## Funding

None.

## Disclosures

None.
